# Experience of Caring as Source of Abductive Reasoning in Nursing: a Pragmatic Vision

**DOI:** 10.17533/udea.iee.v40n3e07

**Published:** 2023-02-10

**Authors:** Virginia Inés Soto Lesmes, Jaime Alberto Ramírez Niño, Luz Stella Bueno-Robles

**Affiliations:** 1 Nurse, PhD. Full Professor. Universidad Nacional de Colombia - at Bogotá - Colombia. Faculty of Nursing. Email: visotol@unal.edu.co Universidad Nacional de Colombia Universidad Nacional de Colombia Faculty of Nursing Bogotá Colombia visotol@unal.edu.co; 2 Nurse, Master’s. Professor, Direction of Nursing, Universidad ECCI. Email: jramirezni@ecci.edu.co Universidad ECCI jramirezni@ecci.edu.co; 3 Nurse, PhD. Associate Professor, Faculty of Nursing. Universidad Nacional de Colombia - at Bogotá. Colombia. Email: lsbuenor@unal.edu.co Universidad Nacional de Colombia Faculty of Nursing Universidad Nacional de Colombia Bogotá Colombia lsbuenor@unal.edu.co

**Keywords:** nursing care, philosophy nursing, clinical reasoning, nursing theory, nursing practice., cuidado de enfermería, filosofía en enfermería, razonamiento clínico, teoría de enfermería, enfermería práctica., cuidados de enfermagem, filosofia em enfermagem, raciocínio clínico, teoría de enfermagem, enfermagen práctica.

## Abstract

The aim of this reflection article consists in proposing a methodology that makes visible the epistemic practice through abductive reasoning for the generation of knowledge from an experience of caring. For such, the work describes the connections between the science of nursing and inter-modernism, develops the idea of the nursing practice as source of knowledge, and defines the components of abductive reasoning for the practice. Finally, the work presents an academic exercise developed within the framework of the assignment *Evaluation of the theory for research and practice* in the PhD program in nursing at Universidad Nacional de Colombia on how a theory was developed from a situation of care and its scientific usefulness upon generating in patients a sense of fullness in their health and in nursing professionals, satisfaction with their work.

## Resumen

El objetivo del presente artículo de reflexión consiste en proponer una metodología que visibilice la práctica epistémica mediante el razonamiento abductivo para la generación de conocimiento a partir de una experiencia de cuidado. Para ello, se describen las conexiones entre la ciencia de enfermería y el intermodernismo, se desarrolla la idea de la práctica de enfermería como fuente de conocimiento y se definen los componentes del razonamiento abductivo para la práctica. Finalmente, se presenta un ejercicio académico desarrollado en el marco de la asignatura *Evaluación de la teoría para la investigación y la práctica* del programa de Doctorado en Enfermería de la Universidad Nacional de Colombia sobre cómo se desarrolló una teoría a partir de una situación de cuidado y su utilidad científica al generar en el paciente una sensación de plenitud en su salud y en el profesional de enfermería, satisfacción con su labor.

## Resumo

O objetivo deste artigo de reflexão é propor uma metodologia que torne visível a prática epistêmica por meio do raciocínio abdutivo para a geração de conhecimento a partir de uma experiência de cuidado. Para tanto, descrevem-se as conexões entre a ciência da enfermagem e o intermodernismo, desenvolve-se a ideia da prática de enfermagem como fonte de conhecimento e define-se os componentes do raciocínio abdutivo para a prática. Por fim, apresenta-se um exercício acadêmico desenvolvido no âmbito da disciplina *Avaliação da teoria para pesquisa e prática* do programa de Doutorado em Enfermagem da Universidade Nacional da Colômbia sobre como se desenvolveu uma teoria a partir de uma situação de cuidado e sua utilidade científica gerando no paciente uma sensação de plenitude em sua saúde e no profissional de enfermagem, satisfação com seu trabalho

## Introduction

It is acknowledged that the level of jurisdiction of a profession on the respective practice is related with the presence of abstract thought and use of theories in the practice.(1) Although nursing is recognized as the profession of care in the experience of human health, the jurisdiction over its own practice is limited.(1,2) Fawcett J *et al*.,(3) recognize that, without its own theoretical and conceptual knowledge, the nursing practice fails to be at the forefront of the discipline because it does not comply integrally with the mission of satisfying the demands de health care de las personas. In this sense, Reed insists on the need to demonstrate that a theory can emerge from a specific situation in nursing care.(4) Frequently, nursing professionals act according to their best judgement, establishing simultaneous connections between their profound observations and what patients themselves reveal based on existing theories to explain an experience of caring, that is, epistemic practice.(4-8) 

### Nursing science and inter-modernism

Inter-modernism permits expanding the view of the world between modern and postmodern postures because it does not abandon the useful categories of science, but rather creates alternatives to think a philosophy that facilitates the development of nursing knowledge through the care practice. This perspective promotes an epistemology that associates science with the practice to create knowledge, which is developed, above all, in pragmatic and modifiable manner, given that upon reflecting on the nursing processes that compromise the population’s wellbeing, it is found both experience and doing.(5) In turn, the science of nursing is defined as the systematic investigation of health and healing processes between humans and their environment, so that it integrates diverse sources of knowledge, like ethics, science, and the nursing practice.(9) Thus, the practice influences positively on the development of knowledge, not only in the context of discovery, in which nursing professionals and scientists are inspired and formulate theoretical ideas to study a problem; but also in the context of justification, epistemic area that permits nursing professionals and scientists to systematically examine, test, and refine their theories.(9) This configuration entails theories requiring sufficient empirical heritage and which, in turn, are useful from scientific evidence for nursing professionals, considered *situated connoisseurs.*(10)

### Pragmatism, abductive reasoning, and nursing

Pragmatism is not concerned with discovering or demonstrating absolute truths, instead, it is interested in how actions create possibilities that explain best the problems of human daily life. Experiences are part of reality, but it has the potential to change and be influenced by the context upon demonstrating certain degree of utility for science and society.(11) Because nursing is of pragmatic nature, it could be stated that the development of this discipline is also pragmatic. In other words, the nursing setting transcends from a context of theoretical application to one of generation of knowledge.(4) The nursing practice is an essential and inexhaustible source of critical judgment; so, by observing surprising phenomena, it is possible emerge from pre-established frameworks. In effect, thus, new ways are discovered of analyzing phenomena characteristic of the discipline and conforming an abductive path for the construction of knowledge. .(4) .(12) 

Abductive reasoning is a form of creative inference and an explicative method, which implies not only the integration, but also the argumentation of ideas, whose purpose consists in constructing new knowledge from progressive comprehension of how truth can be determined.(13,14) Assimilating abductive reasoning to the nursing practice is a recourse that requires being studied to propose initiatives about how the best strategies that demonstrate the usefulness of care in practice should work.(13) The purpose of this strategy lies in solving the needs of the subject of care and exercise with greater solvency the discipline itself. Likewise, the theoretical thought of nursing is based on observations and profound relations with the subjects of care, articulated with evidence available from the disciplinary theory, although generating hypotheses supported on the practice without a pre-existing theoretical support.(4,5) So it is in practice that the usefulness of the abductive process transcends and permits defining the best care for the patient, upon obtaining broader and deeper understanding of the nursing care inquiry, which is validated simultaneously in its practice, with some connections of the existing theory. .(4) .(15) 

The nursing practice is not only direct source of knowledge, but is also anchored to theoretical thought through diverse forms of reasoning, which can be deductive, inductive, or abductive; the last is the focus of interest for our analysis.(4) .(5) From abductive reasoning, nursing professionals integrate observation, interaction with patients, theory, experience, and patterns of existing knowledge to explain an care experience. .(6) .(8) Similarly, it proposes and evaluates healing actions in supported manner, producing knowledge for action.(12) From this epistemological notion of nursing care, the aim of this article consists in proposing a methodology that makes visible its epistemic practice through abductive reasoning for the generation of knowledge from a care experience. 

### Components of abductive reasoning for the nursing practice

Abductive reasoning is understood as the faculty of examining a set of facts and permitting for those events to imply a theory, for which is considered accumulated experience, given that the theory cannot be generated without a prior context or a surprising nursing phenomenon.(14) From the approaches of the classics Peirce and Dewey, referenced by Deering(11) and Moscoso(14) allowed to build the components of abductive reasoning so that they could be applied and characterized to nursing practice because they can occur simultaneously or in different moments; however, it is necessary that all occur in the same situation. These are defined briefly in the following:

*Attitude*. The nurse applies in a care situation values and behavioral norms based on knowledge; besides, establishing a mental and physical commitment with the subjects of care.(4)

*Observation.* It is the means by which the nurse observes closely existing, non-routine problems of the practice and gathers data on significant and unexplainable details in a given care situation. From this situation, and interpretation emerges.(4) .(5).

*Interpretation*. This phase appeals to the *comprehensive interpretation* method, according to which in the nursing practice it is necessary «to really include the people involved in the problem situation in question and work to construct a narrative that gives meaning to the experience and proposes ways of improving the situation».(11)

*Prior knowledge*. The nurse applies nursing knowledge to a specific care situation, namely: metaparadigms, visions of the world, concepts and theories specific of the discipline, as well as the knowledge of the human being in its biological, psychological, and social dimensions. This is modified as the nurse-patient relation is strengthened. .(4) .(2)

*Clinical skills and abilities.* This component permits obtaining one’s own clinical judgement and jurisdiction in the decisions in the nursing practice. Likewise, it implies not only the development of psychomotor and interpersonal abilities, but also a mental and physical commitment with technology, with human beings, and with other professionals in the practice setting.(4) Skills and abilities are manifested whenever a specific care situation takes place. 

*Construction of initial hypotheses.* This phase postulates a potential explanation that emerges spontaneously when weighing a phenomenon of interest for the nurse under a concrete circumstance. .(14) .(5) 

*Construction of explicative hypotheses.* With this component, it is possible to extract the likely consequences derived from the care action and, from the initial hypotheses, predictions are proposed, which are taken to action and are compared with the results of the experiments. Thus, the hypotheses that obtain the best result will be preferred over the alternative ones.(14)

*Construction of a new theory.* Initially, a path was used that guided the strategy to organize the knowledge constructed.(1) The theory is created the moment the nursing intervention is conducted through a complex process —but still insufficiently understood— of reflection on said intervention.(5) Upon evidencing said process and the epistemological analysis of the act of caring, the experience acquires scientific nature and, hence, enters the terrain of theory. 

*Result*. The result on health is a component that indicates the level of success achieved in the patient, that it, checks to see if what was intended has been achieved with the care interventions during the care process.(16) It also corroborates that the new theory constructed permits strengthening the disciplinary development of nursing.(3) 

### Experience of caring

To analyze a given experience of caring from abductive reasoning, initially, it is fitting to recreate a real and significant fact that proceeds from said experience through a narrative that accounts for the interactions and actions present in it. In this sense, the following transcribes a story reconstructed during the second semester of 2019, which shares a care interaction within the context of an intensive care unit in a Colombian health institution. 

### Narrative: The window toward the language of the heart

«The end-of-year festivities welcomed us in that chaotic place called ICU*.* Mr. Aries and I faced spiritual loneliness: he from his health situation —impaired physical mobility, body entrapment secondary to degenerative and progressive hell (*denominated amyotrophic lateral sclerosis*), faces of sadness, tears, always lost gaze and insomnia—, aggravated by family abandonment; *and I, far from my family and feeling as my own Mr. Aries’s loneliness*. His frail, weak and immobile body evidenced a significant deterioration in its domain of perception and cognition, with imminent risk of cessation of spontaneous breathing and cardiovascular activity, which required my full attention. 

A meeting of gazes was enough to establish an extrasensory connection and touch emotional fibers that immediately generated an eruption of feelings. We were two souls in one, who through silence called each other to keep each other company within a storm of silent pleads that sought to link two experiences*.* His increased heartbeat and breathing told me something unrelated to his clinical condition. Little by little, that connection merged two experiences, as if each opened a window from their heart to allow the other to enter and elucidate the essence of two beings as if they were one. A horizon began to be drawn in the only window of that cubicle, where the gaze of Mr. Aries and mine converged; it was there that I could feel what two trapped souls needed.

He needed me to be there, so I dragged a chair over that was nearby and sat by his side —while I remembered that at some point in my life, I had done it with a loved one who was sick and that the only thing he wanted at that moment was my company—. Silence encapsulated that instant and harmonized a moment in which two beings with distinct sufferings joined their gaze towards a new dawn, a new beginning, we left behind the hardships that overwhelmed them and traced new goals towards a comforting and encouraging future. So, we felt that, after that deep reflection, we needed to happily invigorate that moment, so it occurred to me to look in my desk for an old clock-radio that on many occasions entertained me even during the most complex shifts; I placed it on Mr. Aries’ table and turned it on. 

It was then that each note and melody stimulated emotional vibrations; when silent laughter and bizarre dances flowed in a *spiritual revelry* in which the only guests were Mr. Aries and myself. We drank the nectar of absurd happiness, which little by little intoxicated our hearts; we were celebrating, more than the end of the year, the meeting of two beings and the fusion of two souls in a feeling of friendship that radiated enough energy to adorn that time in the intensive care unit.

Those few minutes shook my perspective on nursing care. So, I felt that said connection had a meaning for me and for Mr. Aries because there I was able to transition from a clinical nursing focused on hemodynamic, neurological and respiratory monitoring; on the administration of medications and on invasive procedures, to nursing of the soul and the spirit, whose unintelligible languages generated such energy that drove care in unimaginable ways. This made that experience —perhaps common for any other— to mean a cognitive and spiritual transcendence for Mr. Aries and for me. Sometimes, when I share time with my family, I remember Mr. Aries and cherish each minute; well, I understand that a material transaction is not necessary to help someone when feelings are transmitted with the heart even in life’s most simple actions ».

Table 1, in response to that proposed by Reed(4) on the imperative of innovating in tools that demonstrate how knowledge is produced for the practice, presents the components of abductive reasoning that are implicit and explicit in the narrative. Furthermore, these components evidence that it is possible to generate useful theories to solve the patient’s health needs.

**Table 1:** Components of abductive reasoning from the narrative: *The window toward the language of the heart*

Component	Evidence from the narrative
Attitude	«The end-of-year festivities welcomed us in that chaotic place called ICU. Mr. Aries and I faced spiritual loneliness: he from his health situation (…)aggravated by family abandonment; and I, far from my family and feeling as my own Mr. Aries’s loneliness (…)».
Observation	«(…)impaired physical mobility, body entrapment secondary to degenerative and progressive hell (denominated *amyotrophic lateral sclerosis*), faces of sadness, tears, always lost gaze and insomnia—, (…)». «(…) His frail, weak and immobile body evidenced a significant deterioration in its domain of perception and cognition, with imminent risk of cessation of spontaneous breathing and cardiovascular activity, which required my full attention (…)».
Interpretation	«A meeting of gazes was enough to establish an extrasensory connection and touch emotional fibers that immediately generated an eruption of feelings. We were two souls in one, who through silence called each other to keep each other company within a storm of silent pleads that sought to link two experiences (…)».
Prior knowledge: The nursing professional enters the care situation from an interactive-integrative perspective. The narrative reflects not only the patient’s physical condition, but also the feelings, emotions, and how Mr. Aries’ health condition was permeated by the context: *family abandonment* and *the chaos in the ICU*. The professional’s expertise permits their considering that through the nurse-patient relation they can transcend to a unitary-transformative vision.	Metaparadigm: *Human being*: «(…) —impaired physical mobility, body entrapment secondary to degenerative and progressive hell (*denominated amyotrophic lateral sclerosis*), faces of sadness, tears, always lost gaze and insomnia— (…)». «(…) His frail, weak and immobile body evidenced a significant deterioration in its domain of perception and cognition, with imminent risk of cessation of spontaneous breathing and cardiovascular activity, which required my full attention (…)». *Environment*: A health situation aggravated by the family abandonment and chaos in the ICU. *Health*: Initially, the physical, mental, and emotional health condition of Mr. Aries was critical, given that it required satisfying the needs his body demanded. But, throughout the care interaction, this concept evolves toward a sense of fullness and wellbeing of both: «It was then that each note and melody stimulated emotional vibrations; when silent laughter and bizarre dances flowed in a *spiritual revelry* in which the only guests were Mr. Aries and myself. We drank the nectar of absurd happiness, which little by little intoxicated our hearts; we were celebrating, more than the end of the year, the meeting of two beings and the fusion of two souls in a feeling of friendship, radiating enough energy to adorn that time in the intensive care unit» *Nursing*: The patient’s needs are identified; creative, sensitive, unique, unrepeatable, and transcendent care is provided toward the wellbeing of the nurse-patient relation: «He needed me to be there, so I dragged a chair over that was nearby and sat by his side (…), we left behind the hardships that overwhelmed them and traced new goals towards a comforting and encouraging future. So, we felt that, after that deep reflection, we needed to happily invigorate that moment, so it occurred to me to look in my desk for an old clock-radio that on many occasions entertained me even during the most complex shifts, I placed it on Mr. Aries’ table and turned it on. It was then that each note and melody stimulated emotional vibrations; when silent laughter and bizarre dances flowed in a *spiritual revelry* in which the only guests were Mr. Aries and myself.
Construction of initial hypothesis	In the care relation, nurses apply knowledge from the support of nursing theories, clinical experience in managing human beings in critical health situation, and their own history to provide direct care to a person with progressive physical deterioration and family abandonment.
Construction of explicative hypothesis	The nurse notes that the progressive physical deterioration of the human being to whom care is being provided leads him to feeling trapped in his body; a feeling *aggravated* by the loneliness caused by *family abandonment*.
	The limitation of the human being subject of care to communicate verbally induces to a rapid and creative interaction by the nurse with him through the gazes that facilitate care actions.
	The exchange of gazes loaded with emotions generates mutual feelings to recognize that both experienced loneliness and suffering, although in different ways.
	The gazes transcend from the physical to the spiritual to connect both mutually and permit the *Dasein* or *being there* and *helping the other*. Thus, it is possible to grow through the interaction and achieve wellbeing.
	Unintelligible languages create an energy that drives the nurse to provide care in an unimaginable way.
	The nurse intentionally accompanies the subject worthy of care by sitting next to him, while both maintain the gazes and listen to each other in silence.
	Knowledge on the effect of music as a neurological stimulus is a means that allows the nurse to be emotionally present in the other.
	The interaction transforms the nurse and the subject worthy of care in a *spiritual conjunction* that transcend into wellbeing.

Source: Elaborated by the authors from analyses of the nursing situation.

### Path to organize knowledge

**Figure 1:**
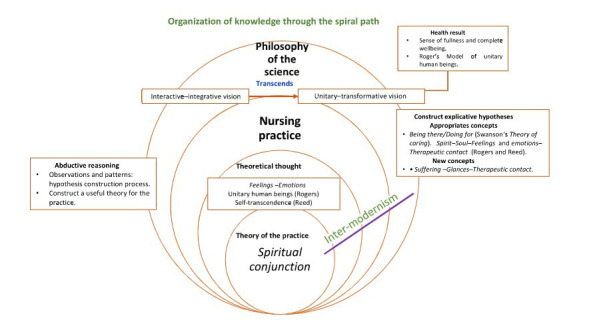
Path for the new *Spiritual conjunction* theory of the practice

Source: Elaborated by the authors from Reed’s presumptions.(1)

This exercise adopts the scheme of a path for the production of nursing knowledge that contributes to the epistemological development for the discipline (Figure 1). It is based on a knowledge creation process that, although not definitive, represents the natural, the dynamic and, at times, the disorganized aspects of nursing practice processes, science and theorization. The primary objective of this spiral path is to promote both thought and theory among nursing professionals from the following question: what does the development process of what has been called nursing knowledge consist of? 

### Construction of the theory

The process of knowledge creation, quite useful for the practice, describes and explains the theoretical concepts that emerged during the care situation object of study herein. These concepts generated the *Spiritual conjunction* theory (Table 2). 

**Table 2:** Concepts of the Spiritual conjunction theory

Concept	Theoretical definition	Representation in the nursing situation
Spiritual conjunction (core concept). Proposition: the expressions of the immaterial component of the human being are triggered by shared suffering and are exposed through portal of the gazes in which the souls of the nurse and the subject of care reach the conjunction, which allows the free flow of feelings and emotions permeated by melodies, presences and intangible contacts.	It is the fusion of human essences (soul), where needs are satisfied that go beyond the physical and the emotional, which achieves a mutual energetic balance connoted by a feeling of fullness —definition derived from the narrative object of study—.	«A meeting of gazes was enough to establish an extrasensory connection and touch emotional fibers that immediately generated an eruption of feelings. We were two souls in one, who through silence called each other to keep each other company within a storm of silent pleads that sought to link two experiences (…)».
Visions (secondary concept)	These are the means to express the human essence —definition derived from the narrative object of study—.	«Little by little, that connection merged two experiences, as if each opened a window from their heart to allow the other to enter and elucidate the essence of two beings as if they were one.».
Soul (secondary concept)	It is the human being’s essential energetic field—definition derived from Roger’s Conceptual model of unitary human beings—.	«(…) that connection merged two experiences (…)».
Feelings and emotions (secondary concept)	Has to do with the human being’s energetic fluctuations —definition derived from Reed’s Theory of self-transcendence, and from the narrative object of study—.	«(…) eruption of feelings (…) a storm of muted pleads (…)».
Being there/Doing for (secondary concept)	Recognized as the intangible presence that implies both verbal and non-verbal messages and simultaneously enables a mutual therapeutic action —definition derived from Swanson’s Theory of caring and from the nursing situation object of study—.	«I dragged over a chair that was nearby and sat by his side (…). Silence encapsulated that instant and harmonized a moment in which two beings with distinct sufferings joined their gaze towards a new dawn, a new beginning, we left behind the hardships that overwhelmed them and traced new goals towards a comforting and encouraging future».
Therapeutic contact (concept or mediating factor)	Refers to any contact between the nursing professional and the subject of care, whose meaning transcends physical contact and whose purpose is to achieve mutual spiritual growth —definition derived from Roger’s conceptual model of unitary human beings, Reed’s Theory of self-transcendence, and from the narrative object of study—.	«A meeting of gazes was enough to establish an extrasensory connection and touch emotional fibers that immediately generated an eruption of feelings. We were two souls in one, who through silence called each other to keep each other company within a storm of silent pleads that sought to link two experiences».
Melody (concept or mediating factor)	It is the environment based on the dispersion of sound waves that contributes to a therapeutic contact —definition derived from the narrative object of study and from Reed’s Theory of self-transcendence—.	«(…)so, it occurred to me to look in my desk for an old clock radio that on many occasions entertained even during the most complex shifts, I placed it on Mr. Aries’ table and turned it on (…)». Mr. Aries and I faced spiritual loneliness: he from his health situation —impaired physical mobility, body entrapment secondary to degenerative and progressive hell (denominated amyotrophic lateral sclerosis), faces of sadness, tears, always lost gaze and insomnia—, aggravated by family abandonment; and I, far from my family and feeling as my own Mr. Aries’s loneliness.
Suffering (triggering or initial concept)	Considered as an emotional state that transcends the physical and emotional ailment caused by their health situation and family abandonment —definition derived from the narrative object of study—.	«Mr. Aries and I faced spiritual loneliness: he from his health situation —impaired physical mobility, body entrapment secondary to degenerative and progressive hell (denominated amyotrophic lateral sclerosis), faces of sadness, tears, always lost gaze and insomnia—, aggravated by family abandonment; and I, far from my family and feeling as my own Mr. Aries’s loneliness».

Source: Elaborated by the authors from analyses of the nursing situation.

**Figure 2:**
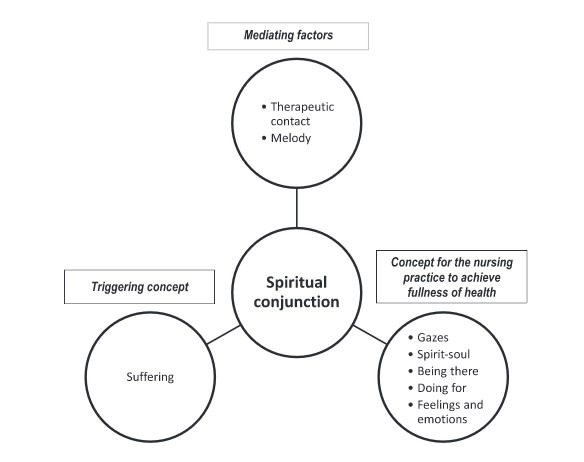
*Spiritual conjunction* theory of the practice

Source: Elaborated by the authors from analyses of the nursing situation

The concepts for action, in terms of the nursing practice according to the nursing situation analyzed to achieve fullness in health, were *Visions*; *Soul*; *Feelings and emotions*; *Dasein* or *Being there* and *Doing for*.(17,18) Likewise, two mediating factors emerged, namely: *Melody and Therapeutic contact*. From the identification of these components, the scheme was established in which the following two factors prevail: *Absence of hierarchy* and *Presence of simultaneity*, whose purpose consisted in constructing the theory so that it explained the reality experienced from a unitary-transformative vision (Figure 2). 

## Discussion

Karlsen *et al*.,(13) approached abductive reasoning as a sort of *starting point* for the research process in the following four steps: preconception of the phenomenon and construction of preliminary hypotheses; empirical confirmation; hypothetical evaluation; and construction of the theory, which can contribute new knowledge to the nursing practice. Nevertheless, this proposal seeks to approach abductive reasoning from a practical experience of care in everyday life, which in addition to the steps proposed for research, also includes in its components the attitudinal, capacity for observation, clinical skills and abilities, and report of health results. Of course, the new theory constructed is not generalizable; it is only valid to solve the health needs of the subject of care: Mr. Aries, in this specific situation. 

## Conclusion

Each experience of caring is not only complex, but also unique and unrepeatable, given the nature of the interaction between the nursing professional and patient, which emerges in a particular context and influences on the situation. However, sometimes this complexity of the practice is not explained or solved with the knowledge constructed until now by nursing, that is, with the theories available. 

Presenting this academic exercise through an experience of caring constitutes a methodological proposal to construct useful theories and make the practice visible through abductive reasoning. It will facilitate, on one part, the use of theoretical thought in concrete scenarios and, moreover, tools to teach it to nursing students to enhance the quality of care. It is necessary to apply this proposal in the different care environments to nourish its validity.
